# Necrotic Ileal Diverticulum With Features Mimicking Meckel’s Diverticulum: A Diagnostic Challenge

**DOI:** 10.7759/cureus.109387

**Published:** 2026-05-21

**Authors:** Brandon Alejandro Lopez-Alanis, Luis A Huerta-Diaz, Luis Zorrilla-Nuñez, Gerardo E Muñoz-Maldonado

**Affiliations:** 1 General Surgery, Hospital Universitario "Dr. José Eleuterio Gonzalez", Monterrey, MEX

**Keywords:** acute abdomen, bowel ischemia, exploratory laparotomy, ileal diverticulitis, intestinal obstruction, meckel diverticulum, pneumatosis intestinalis, small bowel diverticulosis

## Abstract

Small bowel diverticulosis is an uncommon condition that most frequently involves the duodenum and jejunum. In contrast, ileal diverticula are rarely encountered and may present a diagnostic challenge, particularly in the setting of an acute abdomen.

We report the case of a 60-year-old male with no prior surgical history who presented with a 48-hour history of abdominal pain, nausea, vomiting, and obstipation. Contrast-enhanced CT revealed dilated small bowel loops with a transition point in the ileum, raising concern for small bowel obstruction. The patient underwent emergency exploratory laparotomy. Intraoperative findings revealed a solitary necrotic diverticulum located approximately 60 cm proximal to the ileocecal valve on the antimesenteric border, initially suggestive of Meckel’s diverticulum. A segmental ileal resection with double-barrel ileostomy was performed.

Histopathological examination demonstrated transmural ischemic necrosis and acute inflammation, without evidence of ectopic gastric or pancreatic tissue. Although smooth muscle fibers were identified, the lesion did not demonstrate the complete mural architecture expected in a true congenital diverticulum. These findings support a diverticular lesion with overlapping features of both Meckel’s and acquired ileal diverticula, highlighting the diagnostic complexity in such cases.

Although rare, ileal diverticula should be considered in the differential diagnosis of acute abdomen. In the presence of imaging findings suggestive of obstruction, prompt surgical intervention is essential. This case underscores the importance of integrating clinical, radiological, intraoperative, and histopathological findings when evaluating atypical presentations of small bowel pathology.

## Introduction

Small bowel diverticulosis most commonly affects the duodenum and jejunum, whereas ileal diverticula are rare and account for only a small minority of cases. The reported prevalence of jejunoileal diverticulosis ranges from approximately 0.2% to 1.3% in autopsy series and 0.3% to 1.9% on contrast studies, with ileal involvement representing only a fraction of these lesions [1-3].

When a diverticulum is identified in the distal ileum, the primary consideration is typically Meckel’s diverticulum, a congenital true diverticulum arising from incomplete obliteration of the vitelline duct [3,4]. In contrast, acquired ileal diverticula represent a distinct pathological process, usually consisting of mucosal and submucosal herniation through points of weakness in the muscular layer [2-5].

Most cases remain asymptomatic [2,5]. However, complications such as diverticulitis, perforation, hemorrhage, or obstruction may occur and can lead to acute surgical presentations [2,5,6].

## Case presentation

A 60-year-old male presented to the emergency department with a 48-hour history of progressively worsening abdominal pain. The pain was described as constant and predominantly located in the lower abdomen, associated with nausea, multiple episodes of vomiting, and constipation for the previous 24 hours. The patient denied fever, hematochezia or melena, unintentional weight loss, anorexia, other constitutional symptoms, and any history of abdominal trauma.

His medical history was unremarkable, with no prior surgeries or known chronic illnesses. He reported chronic alcohol consumption, approximately three beers daily, and active tobacco use, five cigarettes per day.

On physical examination, the patient was hemodynamically stable. The abdomen was distended and rigid, with generalized tenderness and involuntary guarding, more pronounced in the hypogastric region. Bowel sounds were absent. Digital rectal examination revealed an empty rectal vault without a palpable mass or gross blood. Examination of the inguinal and femoral regions showed no evidence of incarcerated or strangulated hernia.

Initial laboratory evaluation revealed leukocytosis with neutrophilia and elevated lactate levels, raising concern for an underlying inflammatory and ischemic process. A complete blood profile with institutional reference ranges is summarized in Table 1.

**Table 1:** Relevant laboratory findings on admission, with institutional reference ranges. Values reflect the initial laboratory evaluation in the emergency setting.

Parameter	Value	Units	Reference range
WBC count	18	K/µL	4.00-11.00
Neutrophils (absolute)	11.3	K/µL	2.00-6.90
Neutrophils	78.2	%	37.0-80.0
Hemoglobin	18	g/dL	12.2-18.1
Hematocrit	55.2	%	37.7-53.7
Platelet count	175	K/µL	142-424
Lactate	3.3	mmol/L	0.9-1.9
Creatinine	2.4	mg/dL	0.6-1.4
Blood urea nitrogen	31	mg/dL	7-20
Glucose	128	mg/dL	60-100
Sodium	130.7	mmol/L	135-145
Chloride	90.7	mmol/L	101-110
Potassium	3.6	mmol/L	3.6-5.0
pH (venous)	7.38	-	7.32-7.43
pCO₂ (venous)	35	mmHg	40-45
HCO₃⁻	20.7	mmol/L	24.0-30.0
Base excess	-3.8	mmol/L	-2.0 to 2.0

Abdominal ultrasound was not performed prior to CT imaging, as contrast-enhanced CT was considered the most appropriate initial diagnostic modality in this clinical scenario. A contrast-enhanced CT scan demonstrated dilated small bowel loops measuring up to 4 cm, with a clear transition point in the mid-ileum (Figure 1). Associated findings included interloop fluid and a small amount of pelvic free fluid. The bowel wall showed preserved enhancement without definitive radiologic signs of transmural ischemia. No significant abdominal lymphadenopathy was identified. The superior mesenteric artery and vein were patent, with no evidence of thrombosis or embolism.

**Figure 1:**
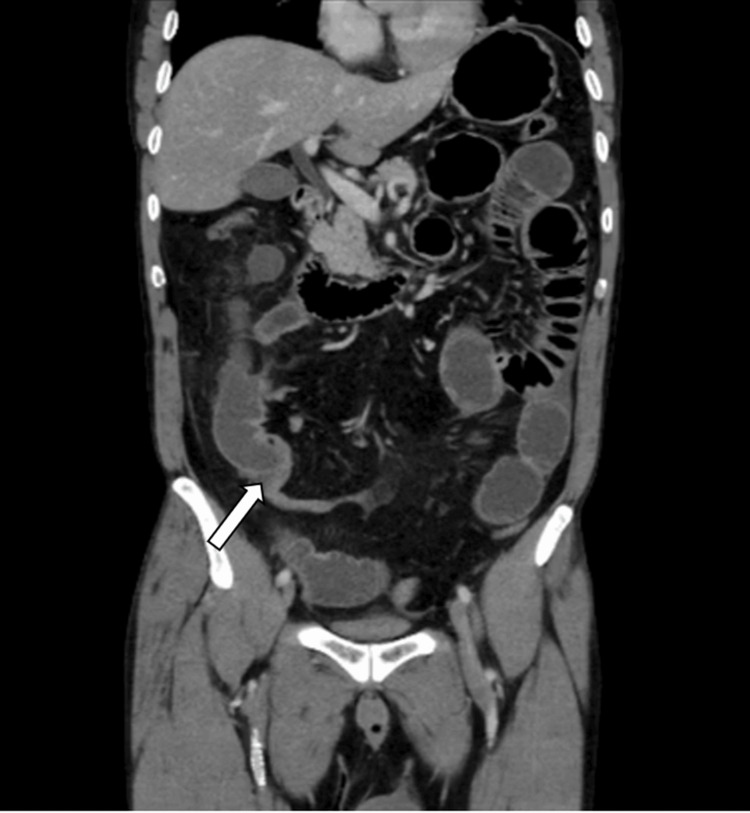
Contrast-enhanced CT scan demonstrating dilated small bowel loops with a transition point in the mid-ileum (arrow), associated with interloop fluid and pelvic free fluid. No definitive radiologic signs of transmural ischemia were identified.

Given these findings, the patient was taken for emergency exploratory laparotomy. Intraoperatively, a solitary diverticulum measuring approximately 10 cm in length, with a base diameter of 2 cm, was identified approximately 60 cm proximal to the ileocecal valve on the antimesenteric border of the ileum and demonstrated clear signs of necrosis, with dark discoloration and fibrinopurulent exudate (Figures 2-3). The affected segment caused mechanical obstruction with collapse of the distal bowel. No evidence of torsion, fibrous bands, or communication with the umbilicus was identified. The superior mesenteric vessels appeared patent, with no signs of thrombus or embolism. No independent vascular pedicle was identified supplying the diverticulum, and no features suggestive of a persistent vitelline artery were observed.

**Figure 2:**
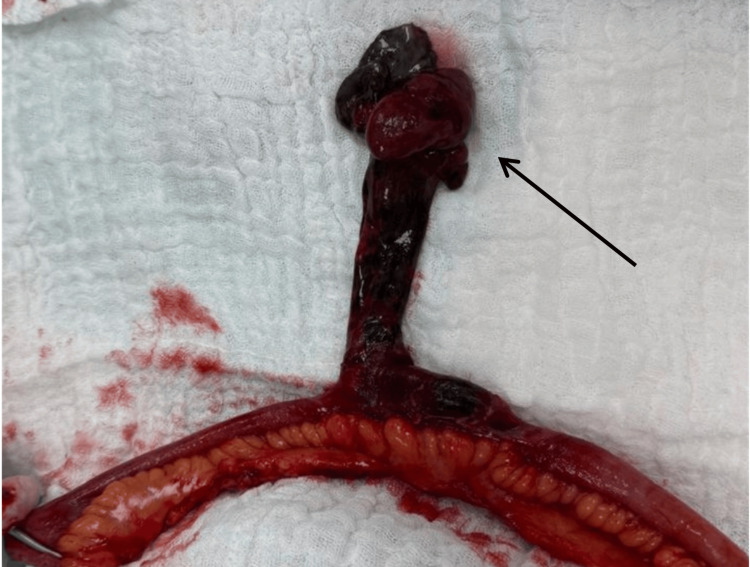
Intraoperative view of the ileal segment demonstrating a solitary diverticulum arising from the antimesenteric border, located approximately 60 cm proximal to the ileocecal valve. The diverticulum shows necrotic changes with dark discoloration and surrounding inflammatory involvement (arrow).

**Figure 3:**
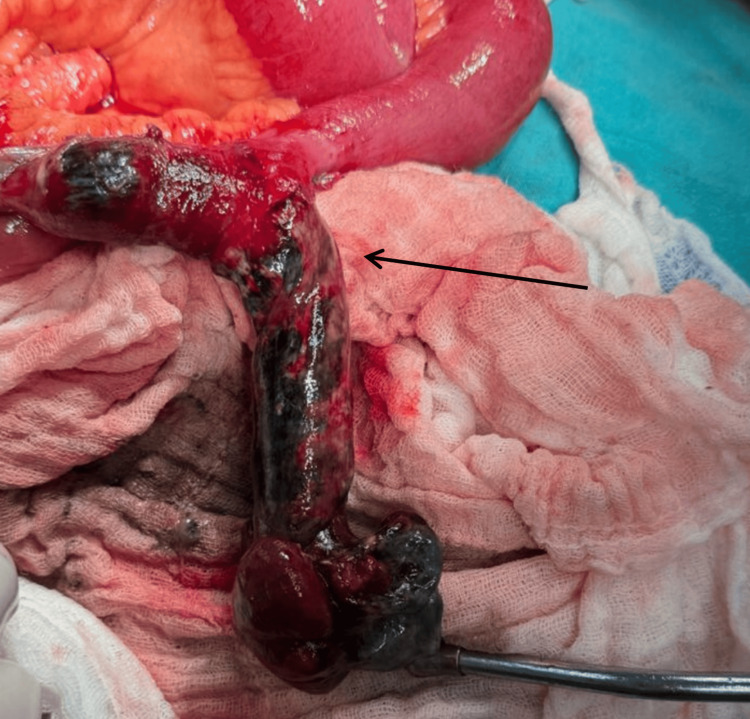
Close-up intraoperative image showing advanced necrosis of the ileal diverticulum (arrow), with a gangrenous appearance and fibrinopurulent exudate. The lesion was responsible for luminal obstruction and distal bowel collapse.

A segmental ileal resection was performed, including 10 cm proximal and 3 cm distal margins. Due to significant bowel edema and peritoneal contamination, primary anastomosis was deferred, and a double-barrel ileostomy was constructed. The remaining small bowel length was approximately 390 cm.

The postoperative course was uneventful. The patient remained hospitalized for three days, tolerated a progressive diet, and was discharged with a functional stoma.

Histopathological examination revealed transmural ischemic necrosis associated with acute inflammatory changes (Figure 4). No ectopic gastric or pancreatic tissue was identified despite extensive sampling. Although smooth muscle fibers were present, the lesion did not demonstrate the complete and well-organized mural architecture expected in a true congenital diverticulum. These findings support a diverticular lesion with features suggestive of an acquired process; however, definitive classification is limited due to extensive ischemic and inflammatory changes, which may obscure the original wall structure.

**Figure 4:**
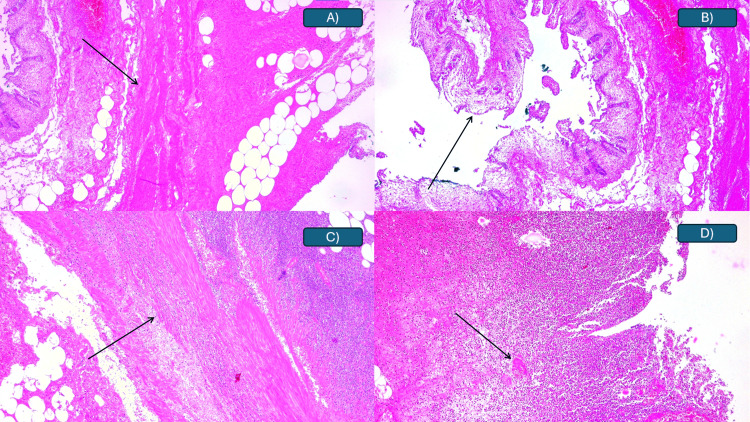
Histopathological findings. (A) Low-power view showing transmural ischemic necrosis (arrow) with loss of normal wall architecture. (B) Disruption and necrosis of the mucosal layer (arrow). (C) Smooth muscle fibers of the muscularis propria (arrows) with adjacent inflammatory changes. (D) Dense acute inflammatory infiltrate consistent with suppurative inflammation (arrow). No ectopic gastric or pancreatic tissue was identified.

## Discussion

This case highlights the diagnostic complexity of ileal diverticula, particularly when anatomical and intraoperative findings closely resemble Meckel’s diverticulum. In the acute setting, surgical decision-making is frequently guided by gross morphology and location. In our patient, the lesion was identified approximately 60 cm proximal to the ileocecal valve and arose from the antimesenteric border, features that are classically associated with Meckel’s diverticulum [3,4]. Additionally, the diverticulum demonstrated a relatively long segment with a narrow base, further contributing to this initial impression.

However, intraoperative assessment did not reveal a fibrous band, communication with the umbilicus, or an identifiable independent vascular pedicle suggestive of a persistent vitelline artery. The superior mesenteric vessels were patent, with no evidence of thrombus or embolism. Despite these findings, the anatomical characteristics remained highly suggestive of a congenital origin.

Histopathological evaluation provided further insight but did not allow for unequivocal classification. While smooth muscle fibers were identified within the wall, the lesion did not demonstrate the complete and well-organized mural architecture expected in a true congenital diverticulum. Instead, the findings were dominated by transmural ischemic necrosis, acute inflammatory changes, and structural distortion, which limited definitive assessment of the original wall layers. Histopathological distinction between Meckel’s diverticulum and acquired ileal diverticula relies on the presence of all layers of the intestinal wall in true diverticula, whereas acquired lesions are typically pseudodiverticula [2-4].

From a pathophysiological standpoint, Meckel’s diverticulum represents a true congenital diverticulum resulting from incomplete obliteration of the vitelline duct, whereas acquired ileal diverticula are formed by mucosal and submucosal herniation through points of weakness in the muscular layer [2-4]. In this case, the overlapping anatomical and histopathological features make strict classification challenging, highlighting the limitations of relying on a single diagnostic modality.

Imaging findings in small bowel diverticulitis may be nonspecific, particularly in early or localized disease. In our patient, contrast-enhanced CT demonstrated small bowel dilation with a transition point and associated interloop fluid and pelvic free fluid, without definitive radiologic signs of transmural ischemia. These findings should be interpreted in conjunction with the clinical presentation and laboratory abnormalities, as radiologic-pathologic correlation is not always direct [6-8].

From a surgical perspective, the management of complicated ileal diverticula, whether congenital or acquired, is largely similar in the acute setting. In the presence of necrosis, perforation, or obstruction, segmental resection remains the preferred approach to ensure complete removal of affected tissue [8-10]. In this case, the presence of transmural necrosis and peritoneal contamination justified resection with adequate margins and temporary diversion.

Overall, this case demonstrates significant overlap between congenital and acquired ileal diverticula, emphasizing that atypical presentations may defy conventional classification. Similar cases have been reported in the literature, reinforcing the importance of integrating clinical, radiological, intraoperative, and histopathological findings when approaching rare and complex cases of small bowel pathology [6,9].

## Conclusions

Although rare, ileal diverticula should be considered in the differential diagnosis of acute abdomen, particularly in patients presenting with small bowel obstruction. In this case, preoperative imaging findings, including the identification of a transition point on CT, provided important diagnostic clues but were not sufficient to establish the underlying etiology.

The distinction between Meckel’s diverticulum and acquired ileal diverticula remains challenging, as demonstrated by the significant overlap in anatomical and histopathological features observed in this patient. While histopathological evaluation is essential, definitive classification may be limited in the presence of advanced ischemic and inflammatory changes.

In the acute setting, prompt surgical intervention remains critical when complications such as obstruction, ischemia, or perforation are suspected. Similar cases have been reported in the literature, reinforcing the importance of integrating clinical, radiological, intraoperative, and histopathological findings to guide management in complex and atypical presentations.
